# Cardiometabolic genomics and pharmacogenomics investigations in Filipino Americans: Steps towards precision health and reducing health disparities

**DOI:** 10.1016/j.ahjo.2022.100136

**Published:** 2022-04-27

**Authors:** Youssef M. Roman, Donna McClish, Elvin T. Price, Roy T. Sabo, Owen M. Woodward, Tesfaye B. Mersha, Nehal Shah, Andrew Armada, Robert Terkeltaub

**Affiliations:** a Department of Pharmacotherapy and Outcomes Science, 410 N 12th Street, Virginia Commonwealth University, School of Pharmacy, Richmond, VA 23298, United States of America; b Department of Biostatistics, 830 East Main Street, One Capitol Square 740, Virginia Commonwealth University, School of Medicine, Richmond, VA 23329, United States of America; c Department of Physiology, University of Maryland School of Medicine, 685 W. Baltimore St., HSF1 580F, Baltimore, MD 21201, United States of America; d Cincinnati Children's Hospital Medical Center, Department of Pediatrics, University of Cincinnati, 3333 Burnet Avenue, MLC 7037, Cincinnati, OH 45229-3026, United States of America; e Division of Rheumatology, Allergy, and Immunology, 1112 East Clay Street, VCU Health Sciences Research Building, Room 4-110, Virginia Commonwealth University, School of Medicine, Richmond, VA 23298-0263, United States of America; f Filipino American Association of Central Virginia, 7117 Galax Road, Richmond, VA 23228, United States of America; g 9-SDVAHCS, Division of Rheumatology, Allergy, and Immunology, USCD School of Medicine, 9500 Gilman Drive, La Jolla, CA 92093, United States of America

**Keywords:** Genetics, Pharmacogenomics, Race, Ethnicity, Cardiometabolic disorders, Gout, Hyperuricemia, Urate, Health disparities, Minorities, Filipinos

## Abstract

**Background:**

Filipino Americans (FAs) are the third-largest Asian American subgroup in the United States (US). Some studies showed that FAs experience more cardiometabolic diseases (CMDs) than other Asian subgroups and non-Hispanic Whites. The increased prevalence of CMD observed in FAs could be due to genetics and social/dietary lifestyles. While FAs are ascribed as an Asian group, they have higher burdens of CMD, and adverse social determinants of health compared to other Asian subgroups. Therefore, studies to elucidate how FAs might develop CMD and respond to medications used to manage CMD are warranted. The ultimate goals of this study are to identify potential mechanisms for reducing CMD burden in FAs and to optimize therapeutic drug selection. Collectively, these investigations could reduce the cardiovascular health disparities among FAs.

**Rationale and design:**

This is a cross-sectional epidemiological design to enroll 300 self-identified Filipino age 18 yrs. or older without a history of cancer and/or organ transplant from Virginia, Washington DC, and Maryland. Once consented, a health questionnaire and disease checklist are administered to participants, and anthropometric data and other vital signs are collected. When accessible, we collect blood samples to measure basic blood biochemistry, lipids, kidney, and liver functions. We also extract DNA from the blood or saliva for genetic and pharmacogenetic analyses. CMD prevalence in FAs will be compared to the US population. Finally, we will conduct multivariate analyses to ascertain the role of genetic and non-genetic factors in developing CMD in FAs. Virginia Commonwealth University IRB approved all study materials (Protocol HM20018500).

**Summary:**

This is the first community-based study to involve FAs in genomics research. The study is actively recruiting participants. Participant enrollment is ongoing. At the time of this publication, the study has enrolled 97 participants. This ongoing study is expected to inform future research to reduce cardiovascular health disparities among FAs.

## 1 Background

Health disparities exist among minority populations in the US, with differences observed in disease prevalence, mortality rates, and responses to medications. These differences are multifactorial with genetic variation explaining a portion of this variability. Additionally, social and lifestyle factors are major contributors to these disparities. The rapidly growing Asian subpopulations are often aggregated under “Asian and Pacific Islanders”. The aggregation of heterogeneous groups with different prevalence of social determinants of health and genomic architectures may be masking the differences in the etiology of the health conditions and the true prevalence of the same health conditions across the different subgroups. [1], [2], [3]. The representation of minority subgroups in research has been also limited. Indeed, most of the research has focused on large racial and ethnic minority categories, which are defined as “White, Black, or African American, American Indian or Alaskan Native, and Asian”. The lack of representation of minority subgroups in research is further aggravating the burden of health disparities and creating knowledge gaps that are hampering our potential to advance the biomedical field to improve public health. [4], [5] FAs are a prime example of an Asian subgroup that has been commonly ascribed as Asian, despite their unique ancestry, social history, and higher prevalence of chronic health conditions than other Asian subgroups. [6] Aggregating the FA and other Asian subgroups under the broader categorization of “Asians” may be leading to health disparities. Thus, the purpose of this study is to provide a focused genomic and pharmacogenomics investigation of the disproportionate prevalence of cardiometabolic risk factors within FAs. Being the first prospective community-based genetic investigation in FAs, this study is expected to add to our growing body of knowledge about the sources of variability of disease risks and the predicted response to drug therapy across different minority groups. The long-term goal of this project is that the inclusion of minority populations in biomedical research will advance our understanding of the genetic and non-genetic heterogeneity of complex traits. This increased diversity in biomedical research will enable researchers to identify tools to reduce the burden of health disparities, promote health equity, and improve overall public health- a roadmap for precision medicine for all.

### 1.1 Health disparities in Filipino Americans

FAs are the third-largest Asian subgroup in the US. Among FAs, the cardiovascular diseases (CVD) death rate is more than 2-folder higher than the general American population. [7], [8] FAs have a substantially higher prevalence of CVD, cardiometabolic disorders, hyperuricemia (HU), and gout compared to non-Hispanic White population with prevalence estimates comparable to Black population. [7] Specifically, using 3-year cross-sectional claims data revealed that 49% of Filipino men are overweight, 32% have type 2 diabetes (T2D), and 38% have ever smoked. [3] Additionally, Filipino men (73%) are at a significantly higher risk of possessing all dyslipidemia subtypes (high LDL-C, low HDL-C, and High TG) compared to non-Hispanic Whites and other Asian subgroups. [3] Other cardiometabolic risk factors such as HU and gout are also common in FAs. Anecdotal reports, case-reports, and cross-sectional studies have demonstrated that FA and Filipino migrants are at higher risk for developing gout compared to non-Hispanics Whites and Filipinos living in the Philippines. [1], [9], [10], [11] Collectively, this differential prevalence in CVD risk factors is a health disparity within the FA community. Additionally, the majority of the cardioembolic-related genomics research in Filipinos has been mostly focused on women and their offspring residing in the Philippines, which hinders the ability to generalize their findings to the overall FA populations and those living in the US. [12]

Designing a multilevel intervention focused on determining the etiology of complex traits, such as cardiometabolic disorders, and tailored to a minority population, such as the FA, will benefit from a community-based genetic research approach. This approach will allow investigators to extensively delineate the role of environment, diet, different lifestyles, and genetics in the onset and management of cardiometabolic disorders and CVD. The ultimate goal of such a research approach is to develop an informed strategy on how to create sustainable and culturally appropriate community-based interventions to the FA community and optimize the national efforts to reduce the burden of health disparities. While there are limited studies focused on health promotion in FA, there are no studies thus far that have used a multilevel approach for disease prevention or health promotion in FA.

The impact of genetic risk factors in developing cardiometabolic disorders is established. [13] While the development of cardiometabolic disorders is polygenic, a few genetic polymorphisms have shown reproducibility across multiple populations with modest effect size and are relatively common in the overall population. For example, the genetic polymorphism rs780094 in the *GCKR* has been implicated in triglyceride metabolism, serum uric acid (SUA) levels, and hyperinsulinemia. [14], [15], [16], [17] The same health conditions also appear to be highly prevalent in FA despite having a low Body Mass Index (BMI). Additionally, the genetic polymorphism rs10811661 in *CDKN2A/B* contributes to the enhanced risk of developing diabetes of up to 40% across different populations. [18], [19], [20] While the effect of genetic polymorphisms in *GCKR*, *APOA5 and APOE* were associated with the levels of lipoproteins in Filipino mothers and their offspring residing in the Philippines, [12] the role of *GCKR*, *APOA5*, *APOE*, *and CDKN2A/B* in the development of cardiometabolic disorders in the overall FA population is unknown. Additionally, there is no genetic information on the frequency of clinically actionable pharmacogenetic variants in FAs, which may contribute to the suboptimal management of cardiometabolic disorders in individuals who self-identify as Filipino. Collectively, genetic information about FA is scarce and this contributes to a knowledge gap.

### 1.2 Study rationale

The first critical need for this study is determining the optimal management of patients at risk for CVD, which has become increasingly challenging given the global rise in the incidence of obesity, diabetes, metabolic syndrome, and hypertension. Thus, the significance of this study is built on addressing two major critical clinical needs in FA. Also, the prevalence of HU and gout, independent CVD and cardiometabolic metabolic risk factors, is significantly rising nationally and globally. [21] With such rapid population growth and high disease burden in the FA community, teasing out the contributing sources of disease etiology will help develop targeted interventions. Also, the selection of drug therapy to manage FA with cardiometabolic disorders is a challenge given the FA's guarded perspectives on receiving medical care and using Western medicines and drugs. Consequently, medication adherence is a significant challenge when such belief systems exist. Thus, conservative strategies towards drug selection represent as an optimal approach for managing any condition within this ancestrally unique population rather than a one-dose-fits-all approach. Indeed, a personalized medicine approach to drug selection, which recognizes an individual's genetic predisposition to cardiometabolic disorders and responsiveness to certain medications, has been shown to improve adherence rates, clinical outcomes (safety and efficacy) and decrease healthcare costs associated with chronic diseases management. [22], [23]

The second critical need is to increase racial and ethnic diversity in biomedical and genetic research. [4], [24] The FA population is a growing immigrant minority in the US but remains minimally represented in biomedical and genomics research. The engagement of minority and immigrant populations, e.g., FA, in genomics and pharmacogenomics research will increase the global benefits of personalized medicine and move us beyond the “the one genotype fits all” approach. With most of the research on Filipinos taking place in the Philippines, engaging FA in research will expand our knowledge of the effect of immigration, different lifestyles, dietary changes, and acculturation on disease risks as well as gene-environment interactions. Therefore, this project will enable the FA community to garner the benefits of genomics research to explain the high prevalence of cardiometabolic conditions within the community and propel the field of precision health moving forward by including populations historically underrepresented in biomedical research. Collectively, the information derived from this project will inform culturally appropriate disease prevention strategies, future community-based intervention research, promote health equity, and reduce the burden of health disparities on the FA community and the healthcare system at large. And with the ultimate goal of using pharmacogenomics in clinical practice to guide patient care, pharmacogenomics studies focused on minority populations are needed [4], [24].

### 1.3 Preliminary data

We showed for the first time that Filipinos residing in Hawaii have a substantially high frequency of the genetic biomarker for developing gout (Table 1) [25]. A limited targeted genotyping of a cohort of pregnant Filipino females (*n* = 190) for the genetic polymorphism rs2231142 G>T in the coding region of *ABCG2*, a major excretory uric acid transporter in the small intestine and kidney [26] showed that the prevalence of the disease risk allele (T) was 46% [25]. The risk variant results in a missense amino acid substitution in the protein (Q141K) causing a 50% reduction in intrinsic transport function [27], coupled with a further reduction in abundance resulting from increased proteasomal degradation [28]. Individuals possessing the Q141K variant demonstrate preferential loss of intestinal excretion of urate [29]. Importantly, the same risk allele has been associated with a 4-fold higher risk of developing HU and gout across different ethnic populations, and with early onset and tophaceous gout in addition to allopurinol resistance [30], [31], [32], [33], [34], [35]. Moreoever, the rs2231142 G>T demonstrates substantial sex differences with stronger associations with SU and gout in European men [26], [36].

**Table 1:** *ABCG2* (rs2231142G>T) frequency in selected populations.

	G allele %	T allele %	P-value
Filipino females (*n* = 190)	54%	46%	Reference
Caucasian (*n* = 113)^a^	88%	12%	<0.001
Han-Chinese (*n* = 45)^a^	71%	29%	0.014
African American (*n* = 2203)^a^	97%	3%	<0.001

a 1000 Genomes Project (Phase III).

ABCG2 also is expressed by hepatocytes and cardiac microvascular endothelial cells, and impaired ABCG2 function leads to increased endothelial IL-8 release in culture [37]. Hence, impaired ABCG2 function and decreased ABCG2 levels appear to promote inflammation. Therefore, we hypothesize that genetic predisposition for HU and gout in FA may partly explain their higher risk for cardiometabolic disorders. Collectively, our preliminary data are consistent with the epidemiology of HU, gout, and cardiometabolic disorders (hypertension, T2D, dyslipidemia, obesity, metabolic syndrome) in FA [1], [38]. However, the replication and validation of our preliminary findings are warranted due to our limited sampling and population demographics.

### 1.4 Hypothesis and scientific premise

It is well-established that immigration and acculturation to the Western world could modulate the risk of developing cardiometabolic disorders among migrant groups, including Filipinos [7]. However, the current knowledge gap is whether nativity and length of residence in the United States have a consequential effect on disease risk, compared with other immigration and acculturation characteristics. Coupled with the dietary and lifestyle modifications due to immigration, the individual genetic predisposition could invariably impact disease liability among Filipinos when exposed to a western diet. And with having one of the highest global frequency of the pleiotropic genetic variant (rs2231142 G>T) in *ABCG2* and other HU and gout risk alleles, it is plausible that Filipinos could be adversely impacted and disproportionately disadvantaged when exposed to a high purine and fat western diet [6], [25], [38]. With our previous work focused on the genetics of granular ethnic minority immigrants such as the Hmong population [[39], [40], [41]], our study premise is that the inclusion of FA in genomics and pharmacogenomics studies will uncover novel information that pertains to the sensitivity and liability to develop selected health conditions and further advance the field of personalized medicine and precision health [4]

### 1.5 Study primary aims

The first aim of this study is to estimate the prevalence of common cardiometabolic disorders in FA, using disease self-report and objective bodily measures. Chronic cardiometabolic disorders of interest include type 2 diabetes, metabolic syndrome, hypertension, dyslipidemia, HU, and gout. Other diseases of interest include chronic kidney, thyroid, and liver diseases. The prevalence of these health conditions in FA will be compared with the overall US population. The second aim of the study is to estimate the prevalence of selected genetic polymorphisms associated with cardiometabolic disorders and kidney disease in FA. This aim will be achieved by conducting targeted genotyping of well-established genetic polymorphisms within selected genes (Table 2). These genes are significantly associated with developing HU, gout, dyslipidemia, and T2D. The prevalence of these disease risk alleles in FA will be compared with other concordant (Southeast Asian subgroups) and discordant (African American and European) populations published data. The third aim of the study is to estimate the prevalence of clinically actionable pharmacogenetic variants in FA. This aim will be achieved by conducting targeted genotyping of guideline-based genetic polymorphisms (Table 3) using CPIC recommendations for cross-population well-validated pharmacogenes. The prevalence of the clinically actionable pharmacogenetic variants in FA will be compared with other populations using large genetic databases, including 1000 Genomes and All of Us.

**Table 2:** List of targeted disease-gene pairs.

Gene (protein)	Protein function	SNP(s)	SNP effect
*ABCG2* (ABCG2)	Major urate efflux transporter expressed in the kidney, liver, and gastrointestinal tract.	rs2231142 (G>T)	Missense variant resulting in Q141K (Glu141Lys) amino acid substitution leading to a reduction in ABCG2-mediated urate transport, urate under-excretion, hyperuricemia, and gout. Also, it could affect the response to allopurinol and lipid-lowering therapies.
*SLC2A9* (GLUT9)	High-capacity urate, fructose, and glucose transporter expressed in liver, kidney, chondrocytes tissues shown to be strongly associated with hyperuricemia and gout.	rs734553 (G>T)	Intronic variant associated with increased susceptibility to gout due to altered transporter affinity for urate.
*SLC16A9* (MCT9)	Monocarboxylic acid transporter protein across cell membranes.	rs1171614 (C>T)	5′ untranslated region (UTR) variant associated with lower serum urate concentrations in individuals of European ancestry
*SLC17A1* (NPT1)	Uric acid transport protein localized at the apical membrane of the renal proximal tubule.	rs1183201 (T>A)	Intron variant reported being in high linkage disequilibrium (r2 = 0.97) with rs1165205, a SNP intronic of SLC17A3 gene found to be related to SU levels and representing a risk factor for gout
*SLC22A11* (OAT4)	An organic anion-dicarboxylate exchanger mediates transport across the apical membrane of the kidney.	rs2078267 (C>T)	Noncoding transcript exon variant associated with lower serum urate concentrations in individuals of European ancestry.
*SLC22A12* (URAT1)	Major urate transporter that mediates the non-voltage dependent exchange of urate for several organic anions, localized at the apical membrane of the renal proximal tubule.	rs505802 (C>T)	An intergenic variant associated with lower serum urate concentrations in individuals of European ancestry.
*GCKR* (GCKR)	Glucokinase regulator associated with metabolic traits such as insulin resistance that may be linked to urate concentrations.	rs1260326 (C>T)	Missense variant that causes a Leu446Pro amino acid substitution within the glucokinase regulatory protein gene. Associated with lower fasting glucose levels and higher risk for elevated triglyceride levels, SUA, and gout (OR = 1.39, 95%CI 1.23; 1.57)
*INHBC* (INHBC)	Member of the transforming growth factor β superfamily that may inhibit activin A signaling, thus affecting a variety of biologic functions including pituitary hormone secretion and insulin secretion.	rs3741414 (C>T)	3′ untranslated region (UTR) variant was reported to interact with OAT4, URAT1, and NTP1 via their C-terminal PDZ motifs and was found to have an association with SU levels.
*RREB1* (RREB1)	Zinc finger transcription factor that binds to gene promoters and regulates calcitonin gene and androgen receptor.	rs675209 (C>T)	Intergenic variant associated with a higher risk for gout in individuals of European ancestry.
*PDZK1* (PDZK)	Scaffolding protein forms a bidirectional urate transport system to maintain balanced urate levels at the apical membrane of renal proximal tubules.	rs12129861 (C>T)	Intergenic variant inked with lower serum urate concentrations in individuals of European ancestry.
*NRXN2* (NRXN2))	Member of the neurexin gene family that serves as a cell adhesion molecule.	rs478607 (G>A)	Missense variant associated with higher serum urate concentrations in individuals of Chinese descent.
*HNF-4A* (HNF-4)	Transcriptional master regulators of *PDZK1* and *ABCG2.*	rs1800961 (C>T)	Missense variant
*APOA5* (APOA5)	Response to statin therapy and circulating LDL-C levels.	rs662799 (A>G)	Upstream variant
*APOL1* (APOL1)	Risk of chronic kidney disease	rs73885319 rs60910145 rs71785313	Missense variants
*APOE* (APOE)	Genetic variants associated with Alzheimer's disease and fasting blood lipids.	rs7412 (C>T)	Missense variant
*CETP* (CEPT)	Involved in the transfer of cholesteryl ester from high density lipoprotein to other lipoproteins. Defects in this gene are a cause of hyperalphalipoproteinemia	rs5882 (A>G)	Missense variant
*LDL-R* (LDL Receptor)	Major cholesterol-carrying lipoprotein of plasma and transports it into cells by endocytosis. Defects in this gene are a cause of familial hypercholesterolemia	rs5930 (G>A)	Missense variant

**Table 3:** Additional polymorphisms of interest.

Gene	SNP	SNP effect
*ABCG2*	rs72552713 (G>A)	Coding sequence variant
*GCKR*	rs780094 (T>C)	Intron variant
*APOE*	rs405509 (C>A)	Upstream variant
	rs7412 (C>T)	Missense variant
*CDKN2A/B*	rs10811661 (T>C)	Upstream variant
*PDZK1*	rs1967017 (A>G)	Synonymous

SNP: single nucleotide polymorphism.

## 2 Methods and design

### 2.1 Sample size calculations

To accomplish the first study aim, the sample size calculation was based on the projected prevalence of gout. We projected and approximate 10% prevalence of gout in FAs and plan to recruit 300 people to test whether the prevalence of gout is different in the FA sample as compared to an assumed known prevalence of 4% in the general population (NHANES 2015–2016), using a 1-sample Chi-squared test (power of 98%). Hypothetically treating the 4% instead as a sample estimate for the reference population, the power would be 82% using a 2-sample Chi-squared test. For the second study aim, using the candidate gene approach for well-established validated genetic polymorphisms to explain the risk of developing cardiometabolic disorders in FA (Table 2), the sample size of 300 participants will provide 80% power to detect a difference in the prevalence of genetic polymorphisms of at least 18% projected in FA, assuming the prevalence in the general population is 10%.

### 2.2 Study population and recruitment

This study will enroll males and females age 18 years or older who self-report as FA with both biological parents considered of Filipino descent and being born or living in the United States. The exclusion criteria of this study are a history of cancer or organ transplant or being a pregnant woman. The community-engagement approach is the founding principle for our recruitment strategy. This study will recruit 300 participants throughout the state of Virginia and nearby regions, including the District of Columbia and Maryland. The study will also recruit participants who respond to posted advertisements around college campuses and major Filipino attraction sites (e.g., churches, clinics, restaurants). Other strategies will include FA organizations' social media platforms, Filipino student organizations, and the annual Filipino American Festival. This strategy aims to ensure that the study includes a diverse and representative FA sample population with exposures to different and varying levels of social determinants of health. This approach will enable us to robustly estimate the true prevalence of the targeted health conditions and their respective allele frequencies in FA. Notably, the recruitment strategy will capitalize on the partnership between the university and the local FA organizations, including the Filipino American Association of Central Virginia (FAACV), the Asian American Society of Central Virginia, and the Philippine Cultural Center of Virginia Beach. These organizations will provide venues to access the FA population for recruitment and to disseminate study information to the FA community. Finally, our recruitment strategy will involve the Filipino Festivals throughout the District of Columbia, Maryland, and Virginia to ensure a true representation of the FA population in our study.

### 2.3 Study procedures

This proposed study has been approved by the VCU IRB (HM20018500). All surveys, data entry forms, and study databases are established using REDCap. Once a participant consents, we will collect demographic and nativity information, level of education, employment and marital status, years in the US, personal and family medical histories using a prespecified disease checklist, and current medication use. Brief dietary and lifestyle surveys will be also administered. Additionally, anthropometric data including height, weight, and waist circumference as well as vital signs including heart rate and three blood pressure readings will be measured and recorded by a trained healthcare professional. Following vital signs assessment, 30 mL peripheral blood samples will be collected to measure basic metabolic chemistry (glucose, HbA1C, SUA, creatinine, fasting lipids, liver enzymes, hs-CRP, TSH). Future use of participants' serum, DNA, and willingness to enroll in future studies will be also ascertained. We will use the analytical lab results to further characterize the prevalence of cardiometabolic disorders. These disorders will include type 2 diabetes, fatty liver index, dyslipidemia, metabolic syndrome, hyperuricemia, and gout. We will carry out a complete blood count (CBC) and extract genomic DNA from white blood cells. Each participant will be thanked for their participation and to receive a $30 gift card. The processing of the samples and biochemical tests will be conducted at a central lab.

DNA quantification and qualification will be analyzed before genotyping. We are customizing and conducting a multiplex design to genotype for approximately 50 single nucleotide polymorphisms (SNPs) across 30 genes using the 12 K Flex PCR system. The selection of gene-disease pairs (Table 2, Table 3) was based on primary literature corroborating the association of the SNPs with the disease. The drug-gene pairs (Table 4) based on genetic-based guidelines. Specifically, we choose these single nucleotide polymorphisms (SNPs) based on population frequencies, genome-wide association studies, and reproducibility across different ethnic and racial groups. We also identified the drug-related SNPs using the published guidelines on the use of genetic information to personalize drug therapy, which is also known as the Clinical Implementation of Pharmacogenetics Consortium (CPIC). The remainder of the SNPs is known to be clinically actionable regarding medications used to manage cardiometabolic disorders and other cardiovascular health conditions. Additional genotyping will follow using the Illumina GDA array with enhanced pharmacogenetic testing. Inflammatory biomarkers including TNF-alpha, IL-1β, IL-2, IL-6, and IL-8 will be measured using multiplexed particle-based flow cytometric assay.

**Table 4:** List of targeted pharmacogenetic polymorphisms.

Gene	SNP	Genotype/haplotype	Select impacted drug
*ADRB1/2*	rs1801252 rs1801253		- Atenolol^a^ - Carvedilol^a^ - Metoprolol^a^
*CYP2C19*	rs4244285	CYP2C19*2	- Clopidogrel - Citalopram - Voriconazole - Proton pump inhibitors
*CYP2C19*	rs4986893	CYP2C19*3	
*CYP2C19*	rs12248560	CYP2C19*17	
*CYP2C9*	rs1799853	CYP2C9*2	- NSAIDs - Warfarin - - Statins
*CYP2C9*	rs1057910	CYP2C9*3	
*CYP2D6*	rs1065852	CYP2D6*10	- Opioids - Antidepressants - Metoprolol^a^ - Carvedilol^a^
*CYP2D6*	rs1135840	CYP2D6*2	
*CYP2D6*	rs28371725	CYP2D6*41	
*CYP2D6*	rs3892097	CYP2D6*4	
*CYP3A5*	rs776746	CYP3A5*3	Tacrolimus
*SLCO1B1*	rs4149056	SLCO1B1*5	- Simvastatin - Atorvastatin
*CYP4F2*	rs2108622	CYP4F2*3	Warfarin
*VKORC1*	rs9923231		Warfarin
*CYP2A6*	rs143731390	CYP2A6*35	Nicotine^a^
*CYP2B6*	rs3745274	CYP2B6*6	- Bupropion^a^ - Efavirenz - Opioids^a^
*G6PD*			- Pegloticase - Rasburicase
HLA-B*15:02			- Carbamazepine - Oxcarbazepine
HLA-B*57:01			Abacavir
HLA-B*58:01			Allopurinol
*UGT1A1*		UGT1A1*28	- Atazanavir - Irinotecan^a^ - Febuxostat^a^

a Indicates a drug with no CPIC guidelines at the time of this publication with respect to the gene involved.

### 2.4 Statistical analysis plan

When applicable, descriptive statistics will be summarized as mean ± SD or median and range for continuous variables such as age, BMI, and other lab values, or frequency and percentages for categorical variables such as sex, cardiometabolic disorders, and other health determinants.

Age- and sex-adjusted and unadjusted overall prevalence and confidence intervals (CI) of hypertension, dyslipidemia, T2D, metabolic syndrome, HU, and gout will be reported. We will estimate HU prevalence and 95% CI defined as SUA ≥ 6 mg/dL in females and SUA ≥ 7 mg/dL males. We will also estimate kidney function using the MDRD equations. Using the case definition of chronic kidney disease (CKD) as an estimated glomerular filtration rate < 60 mL/min/1.73m^2^, we will estimate the prevalence of CKD in FA and assess its association with cardiometabolic disorders using 2-sample Chi-Square or Fisher's exact tests, as appropriate. Prevalence estimates of cardiometabolic disorders, HU, and gout will be compared to the US using publicly available data from NHANES or other studies using Chi-Square tests.

The prevalence and 95% CI of HU, gout, and cardiometabolic disorders risk alleles, and pharmacogenetics variants will be estimated in FA. Further, these estimates will be compared with other populations from the 1000 Genomes Project Phase III using Chi-Square or Fisher's exact tests. Following CPIC guidance on phenotyping, estimated prevalence of normal, intermediate, and poor drug metabolizers in FA will be calculated for each pharmacogene and compared with other populations as above. Associations between HU or gout and the development of cardiometabolic disorders (hypertension, T2DM, dyslipidemia, central adiposity, metabolic syndrome) and CKD will be assessed using 2-sample Chi-Square or Fisher's exact tests. Differences in continuous outcomes such as blood biochemistry test results (uric acid, lipids, glucose levels, etc.) among the three genotypes of each genetic loci will be analyzed using Kruskal-Wallis test or analysis of variance (ANOVA). To estimate the association between variants and phenotypic traits, we will use mixed effects models having age and sex as covariates. The *p*-values for variants will be adjusted for multiple testing using a False Discovery Rate. Adjusted associations with HU or gout will be assessed using generalized linear models by adding additional features as covariates.

To account for the polygenic nature of developing cardiometabolic disorders, individual weighted genotype risk score (wGRS) will be constructed with a weight of 0 when no significant association exists between the risk allele and phenotype of interest (e.g., HU, gout, T2DM, dyslipidemia, etc.). A wGRS of 1, 2 and 3 will be based on odds ratio (OR) values, 1 < OR ≤ 2, 2 < OR ≤ 3, and OR ≥ 3, respectively. The cumulative effect of wGRS on phenotype of interest along with other clinical variables will be examined using logistic regression models. Multivariable log-binomial or log-Poisson regression models will be used to estimate relative risk and predict the effect of genetic and non-genetic factors in developing cardiometabolic disorders using the individual's cumulative wGRS, comorbidities, and select social determinants of health. Receiver Operating characteristic (ROC) curve analysis, including sensitivity, specificity and area under the ROC curve will be used to assess the ability of prespecified health determinants to discriminate between those with and without cardiometabolic disorders.

## 3 Discussion

To our knowledge, this study protocol describes the first community-based study to engage FAs in genetic research. With a community engagement research approach, this study is leveraging the power of community-academia partnership to increase the representation of hard-to-reach populations in clinical research. Indeed, the inclusion of minority populations is warranted to advance the field of precision medicine for the entire population and address established racial health disparities, including CMDs among FAs. Additionally, this ongoing study will quantitively explore some of the effects of acculturation measures among FAs (primarily years lived in the United States) on the risk of developing CMDs while investigating the role of genetics in disease liability.

### 3.1 Dissemination plan

We will conduct the dissemination process using two primary approaches. First, participants who opted into knowing their test results of the biochemical and clinical laboratory assays will receive the results by email and will be encouraged to discuss them with their providers. Second, we will disseminate the aggregate genetic and non-genetic results to the FA community through one of the community's major cultural events. Furthermore, we will present the results of the study at scientific meetings as well as in peer-reviewed journals.

### 3.2 Preliminary results

At the time of this publication, the study has enrolled 97 participants (56% females and 44% males). The mean age of current study participants is 38 yrs. old. Approximately, 50% of study participants reported being non-US born. Nearly all study participants agreed to keep their biological samples for future studies and to be recontacted for future research. Active and passive recruitments are ongoing to increase the representation of different age groups.

### 3.3 Limitations of proposed research

First, this is a small epidemiological cross-sectional study design, so we will not be able to establish causality. Second, information collected about race and ethnicity is self-reported, which can be inaccurate; however, including the parents' race may reduce the bias associated with self-reported race. Third, we are genotyping the study participants for already known genetic variants of selected genes from other racial groups. This approach could limit our ability to identify novel variants within our cohort. However, our study approach remains a viable option to assess how global disease burden alleles compare to FA, which is unknown. Fourth, we acknowledge that developing certain cardiometabolic disorders is polygenic and requires broader genetic coverage, which warrants future genetic analyses. Finally, we recognize that blood collections from community screening events could be challenging in some cases, and some participants may not be willing to give blood. Alternatively, we provide buccal DNA sample collection kits (Oragene OG-500) when no blood collection would be feasible. Though this approach may limit our ability to phenotype the participant, it remains a viable option for genotyping and estimating allele frequency in the population.

## 4 Summary

The inclusion of FAs in clinical and genetic research has been lacking. Therefore, this study represents the first targeted community-based genetic research among FAs. This ongoing study is rooted in the community-engagement research approach to address the high prevalence of CMDs among FAs. The study aims to characterize the disease prevalence of common CVDs risk factors. Additionally, the study will ascertain the prevalence of clinically actionable pharmacogenes that could impact the treatment of CMDs among FAs. Inclusion of hard-to-reach populations in clinical and genetic research is warranted to address health disparities and move us beyond the one genotype fits all approach.

## Abbreviations

CMDs: cardiometabolic disorders
FAs: Filipino Americans
CVDs: cardiovascular diseases
SNP: single nucleotide polymorphism
CPIC: Clinical Pharmacogenetics Implementation Consortium
NHANES: National Health and Nutrition Examination Survey
NSAIDs: non-steroidal anti-inflammatory drugs
SUA: serum uric acid

## Ethics approval and consent to participate

The study (protocol, including quantitative aspects, and trial materials, including patient information and consent form) was reviewed and approved by the Biomedical Research Ethics Committee of Virginia Commonwealth University (Protocol number: HM20018500). Written informed consent to participate is obtained from all study participants before enrollment.

## Consent for publication

Not application.

## Availability of data and materials

Not applicable. Study patient enrollment and data collection is currently ongoing, and no datasets were generated for analysis yet.

## Funding

This is study was internally funded by the Biostatistics, Epidemiology and Research Design (BERD) core of the CTSA (UL1TR002649 from the 10.13039/100006108National Center for Advancing Translational Sciences) and by the CCTR Endowment Fund of 10.13039/100009238Virginia Commonwealth University. The funding body had no role in the design of the study, collection, analysis, and interpretation of data or in writing the manuscript.

## Authors' contributions

YR conceived the study, submitted the grant, received funding, and wrote the manuscript; YR and DC designed the study; DC, EP, OW, RS, TM, NS, AA, and RT revised the manuscript and provided operational feedback on the study design. All authors agreed to the final version of the manuscript.

## Declaration of competing interest

RT reported receiving NIH funding (AR075990, PAG007996) and consulting fees for the following companies: Fortress Bio, Allena, Dyve, and Acquist. The remaining authors declared that they have no known competing financial interests or personal relationships that could have appeared to influence the work reported in this manuscript.
